# Vector Competence for Zika Virus Changes Depending on the *Aedes aegypti*’s Region of Origin in Manaus: A Study of an Endemic Brazilian Amazonian City

**DOI:** 10.3390/v15030770

**Published:** 2023-03-17

**Authors:** Andréia da Costa Paz, Bárbara Aparecida Chaves, Raquel Soares Maia Godoy, Deilane Ferreira Coelho, Ademir Bentes Vieira Júnior, Rodrigo Maciel Alencar, João Arthur Alcântara, Luiza dos Santos Félix, Cinthia Catharina Azevedo Oliveira, Wuelton Marcelo Monteiro, Marcus Vinicius Guimarães Lacerda, Nágila Francinete Costa Secundino, Paulo Filemon Paolucci Pimenta

**Affiliations:** 1 Fundação de Medicina Tropical Dr. Heitor Vieira Dourado, Manaus 69040-000, Amazonas, Brazil; 2 Programa de Pós-Graduação em Medicina Tropical, Universidade do Estado do Amazonas, Manaus 69040-000, Amazonas, Brazil; 3 Instituto René Rachou, Fundação Oswaldo Cruz, Fiocruz, IOCRUZ, Belo Horizonte 30190-002, Minas Gerais, Brazil; 4 Programa de Pós-Graduação em Ciências da Saúde, Fiocruz, IOCRUZ, Belo Horizonte 30190-002, Minas Gerais, Brazil; 5 Instituto Leônidas e Maria Deane, Fundação Oswaldo Cruz, Fiocruz, Manaus 79057-070, Amazonas, Brazil; 6 Department of Pathology, University of Texas Medical Branch, Galveston, TX 77555, USA

**Keywords:** Zika virus, field mosquito population, vector competence, transmission efficiency, infection rate, disseminated infection rate

## Abstract

Zika virus (ZIKV) is transmitted to humans by the infectious bite of mosquitoes such as *Aedes aegypti*. In a city, the population control of mosquitoes is carried out according to alerts generated by different districts via the analysis of the mosquito index. However, we do not know whether, besides mosquito abundance, the susceptibility of mosquitoes could also diverge among districts and thus impact the dissemination and transmission of arboviruses. After a viremic blood meal, the virus must infect the midgut, disseminate to tissues, and reach the salivary gland to be transmitted to a vertebrate host. This study evaluated the patterns of ZIKV infection in the *Ae. aegypti* field populations of a city. The disseminated infection rate, viral transmission rate, and transmission efficiency were measured using quantitative PCR at 14 days post-infection. The results showed that all *Ae. aegypti* populations had individuals susceptible to ZIKV infection and able to transmit the virus. The infection parameters showed that the geographical area of origin of the *Ae. aegypti* influences its vector competence for ZIKV transmission.

## 1. Introduction

Zika virus (ZIKV) is an arthropod-borne virus transmitted via the bite of mosquitoes of the genus *Aedes* (*Stegomyia*). *Aedes aegypti* is the primary vector in tropical and subtropical regions of the globe and is highly adapted to urban habitats [1]. The worldwide distribution of *Ae. aegypti* [2] and its permissiveness to ZIKV contribute to the broad range of regions at risk of Zika fever outbreaks. The disease represents a recurring threat to the worldwide health system [3,4]. In many cities in Brazil, the *Ae. aegypti* index (LIRAa—Rapid Index Survey for *Ae. aegypti*) is a widely used method for the populational control of mosquitoes. LIRAa allows knowing the distribution of the vector in different parts of a city and shows the areas that need greater attention to maintain epidemiological control. However, we do not know whether, besides mosquito abundance (the parameter detected by LIRAa), the susceptibility of mosquitoes to arboviruses could also diverge among districts and thus impact the dissemination and transmission of arboviruses. The understanding of the dynamics of the susceptibility of *Ae. aegypti* to ZIKV in distinct areas of a city is a necessary step to answer these questions.

ZIKV is closely related to other health-relevant arboviruses such as dengue (DENV) and chikungunya (CHIKV). ZIKV was first isolated in the Zika forest of Uganda in 1947 from a sentinel *Rhesus* monkey [5]. The first human cases of ZIKV infection were detected in Uganda and Tanzania in 1952, followed by sporadic cases reported only on the African and Asian continents. The first outbreaks of Zika outside these continents occurred in 2007 in Micronesia and French Polynesia in 2013–2014 [6]. Subsequently, the virus reemerged and spread rapidly across the Americas in 2015 [7,8], causing a significant outbreak in Brazil, where the virus was a cause of neurological disorders such as Guillain–Barré syndrome in adults and microcephaly in neonates [9]. Despite the apparent decrease in cases reported since the last outbreak, ZIKV infection remains a significant global human threat, especially in Latin America [10].

To establish an infection, arboviruses such as ZIKV must overcome multiple barriers within the mosquito for transmission to occur. After digestion of the blood meal, the virus must infect the midgut, disseminate out of the midgut cells, cross the midgut basal lamina to reach the hemolymph, and then infect the salivary glands (SG) to be transmitted to vertebrate hosts through a subsequent blood feeding [11]. The time interval corresponding to the ingestion of the infected blood meal until the vector becomes infectious is the extrinsic incubation period (EIP), which has an average range of 3–14 days for ZIKV [12,13]. All this is related to the vector’s permissiveness to infect and transmit a pathogen, known as called vector competence (VC). Multiple factors can influence VC, and the midgut infection and escape barrier (MIB and MEB) and salivary gland infection and escape barrier (SGIB and SGEB) are the main challenges the virus should overcome to infect and disseminate within the vector [14].

Despite the impact of Zika fever on the global health systems, the vector competence (VC) of field-derived *Ae. aegypti* populations to transmit ZIKV needs to be better understood, especially regarding mosquitoes from geographically close areas such as the districts of a city. Only a few studies have evaluated the ability of local populations of *Ae. aegypti* to be infected and transmit ZIKV in the context of mosquito populational variability in the Americas [7,11,15] and in Old World countries [16,17,18]. The present study assessed the VC and the related parameters such as the infection rate (IR), disseminated infection rate (DIR), viral load (VL), and transmission efficiency (TE) of *Ae. aegypti* populations derived from five geographically distinct areas of Manaus, an endemic Brazilian city. Understanding the patterns of ZIKV infection and transmission among mosquito populations of a municipality may contribute to future vector-borne disease control programs by adding information about mosquito susceptibility to the virus, thus helping to guide the delimitation of areas of epidemiological risk.

## 2. Materials and Methods

### 2.1. Mosquito Collection

About 1200 *Ae. aegypti* eggs were collected from each of the 5 health districts (total of around 6000 eggs) of Manaus, (latitude 3°6′26″ S, longitude 60°1′34″ W, and 39 m above sea level) Amazonas state, using ovitraps that were spatially distributed. Manaus occupies an area of 11,401 km^2^. The city is the capital of the state of Amazonas and has 2,255,903 inhabitants and a population density of 158 inhabitants/km^2^. It is divided into five health administrative regions: North, South, East, West, and Central [19].

Eggs from each health administrative district were allowed to hatch, giving rise to about 550 females as a parental generation. Three successive generations were reared from these females as a single local colony until enough specimens were acquired for the experimental infections. The *Ae. aegypti* mosquitoes were selected and kept at a controlled temperature of 28 °C, 80% relative humidity, and 12/12 light–dark photoperiod. They were subsequently separated and named according to their district of origin. The parental generation of mosquitoes, derived from the field-collected eggs, was checked for ZIKV natural infection using polymerase chain reaction (qPCR) before starting the mosquito colonization to obtain the next generations. A well-established Brazilian colony of *Ae. aegypti* (strain PP-Campos), closed in 1997, was used as the positive control group in this study to validate the infection procedures [2,3,4,14], while the negative controls were the field-derived mosquitoes fed on uninfected blood (Supplementary Figure S1).

### 2.2. ZIKV Culture

Virus stocks of a ZIKV strain that currently circulates in Brazil (ZikaSPH2015) [20] were passaged in an *Aedes albopictus* cell line (C6/36), which was grown in Leibowitz L-15 medium supplemented with 2% inactivated fetal bovine serum (FBS), 20 μg/mL gentamicin, 5 μg/mL amphotericin B, and 200 U/mL penicillin [21]. Virus titration followed the 50% tissue culture infectious dose method [22].

### 2.3. ZIKV Infection of Aedes aegypti

*Ae. aegypti* female mosquitoes (n = 150), aged 3 to 5 days old from the 5 health districts were placed in separate cages and experimentally infected with ZIKV via a membrane feeding assay [4,23]. A blood meal with a virus titer of 1 × 10^6^ PFU/mL was mixed with fresh mouse blood (ratio 2:1) and offered to the mosquitoes as described elsewhere [22]. Mosquitoes were allowed to feed for 1 h on the ZIKV-infective blood meal. Fully engorged females were separated into new cages and maintained on 10% glucose solution *ad libitum* for up to 14 days post-infection (dpi) until the completion of the extrinsic infection period (EIP).

### 2.4. Mosquito Dissection and Viral RNA Extraction

At 14 dpi, 25 mosquitoes from each health district were randomly chosen, anesthetized on ice, and dissected under a stereoscope. Their bodies and head/salivary glands (head/SG) (salivary glands attached to the heads) were individualized and transferred to separate microtubes. The viral RNA was extracted using the QIAamp Viral RNA Mini kit (Qiagen, Hilden, Germany) according to the manufacturer’s instructions and stored at −80 °C. The quantification of ZIKV was carried out using the real-time reverse transcription qPCR with cDNA production using a random primer and M-MLV enzyme and subsequent TaqMan-based qPCR assay (ThermoFisher Scientific, Hampton, VA, USA) [6,23]. The virus was detected using specific TaqMan probes and primers, as described elsewhere [6].

### 2.5. Infection Rate (IR), Disseminated Infection Rate (DIR), Transmission Rate (TR), and Transmission Efficiency (TE)

The intensity of the infection was estimated by determining the number of viral cDNA copies and the viral load (VL) present in the sample. The IR, DIR, and VC were determined for the five *Ae. aegypti* populations. The well-established Brazilian colony of *Ae. aegypti* (strain PP-Campos) was used as a control group. The IR was calculated as the percentage of infected mosquito bodies related to the total number of tested mosquitoes (n = 25). The DIR was the percentage of infected mosquito heads/salivary glands related with the number of infected mosquito bodies [24]. The TR and TE were assessed by evaluating the presence of ZIKV in forced-secreted saliva of infected *Ae. aegypti*. At 14 dpi, 25 mosquitoes from each experimental group were cold-anesthetized and had their legs and wings removed. The proboscis of each one was inserted into a filter tip containing 6 μL of sucrose solution plus fetal bovine serum (1:1) for 30 min. The secreted saliva samples were individually cultivated in C6/36 cells for 5 days as described above, and supernatants were processed via qPCR for ZIKV cDNA copy quantification. The mosquitoes were analyzed for the transmission rate (TR), which corresponds to the proportion of mosquitoes with infected saliva among mosquitoes with disseminated infection, and for the transmission efficiency (TE), which corresponds to the ratio of mosquitoes with the virus in saliva among the total number of mosquitoes tested [25,26].

### 2.6. Ethical Approval

This study was approved by the Committee on Ethical use of Animals at the Tropical Medicine Foundation Dr. Heitor Vieira Dourado, Manaus, AM, Brazil (protocol number 001979/2019.015/FMT-HVD/CEUA).

### 2.7. Statistical Analyses

The body, head/SGs, and saliva viral loads were evaluated among all populations using Kruskal–Wallis one-way ANOVA tests. Mann–Whitney U tests were used to evaluate the significance among viral load medians in each infected mosquito population’s bodies and head/SGs. All statistical analyses were performed in GraphPad Prism software (version 8.0.2, La Jolla, CA, USA), and *p* values ≤ 0.05 were considered statistically significant.

## 3. Results

### 3.1. Infection Rate (IR) and Disseminated Infection Rate (DIR) of the Ae. aegypti Populations

**Figure 1:**
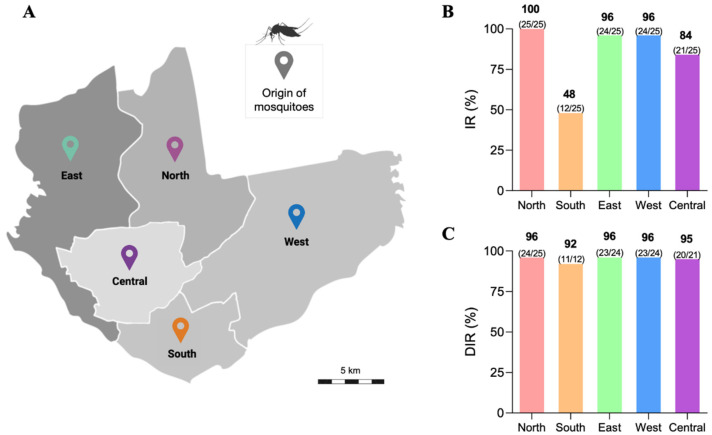
Map of Manaus showing the five districts where the mosquitoes were collected in the field (**A**). Infection rates (**B**) and disseminated infection rates (**C**) of *Aedes aegypti* mosquitoes from 5 districts of Manaus for transmitting Zika virus at 14 days post-infection.

The IRs and DIRs of the 5 *Ae. aegypti* populations were measured at 14 dpi, an incubation time long enough to allow the viral establishment and spreading in the mosquito, thus making it infectious. The results are shown in Figure 1. To facilitate the comparisons of the infection-related rates among the mosquito populations, we assigned arbitrary levels to measure their susceptibility to ZIKV in relation to infectivity (IR) and viral dissemination (DIR). Rates in the 0–40% range were considered low levels; 40–70% intermediate levels; and 70–100% high levels. Considering this classification, the IRs and DIRs values were increased in Northern, Eastern, Western, and Central health districts (ranging from 84 to 100%); and the Southern district had an intermediate IR (48%) and high DIR (92%). No mosquito population had IRs or DIRs rated below 40% (Figure 1).

### 3.2. Viral Load (VL) of the ZIKV via qPCR of Ae. aegypti Populations

**Figure 2:**
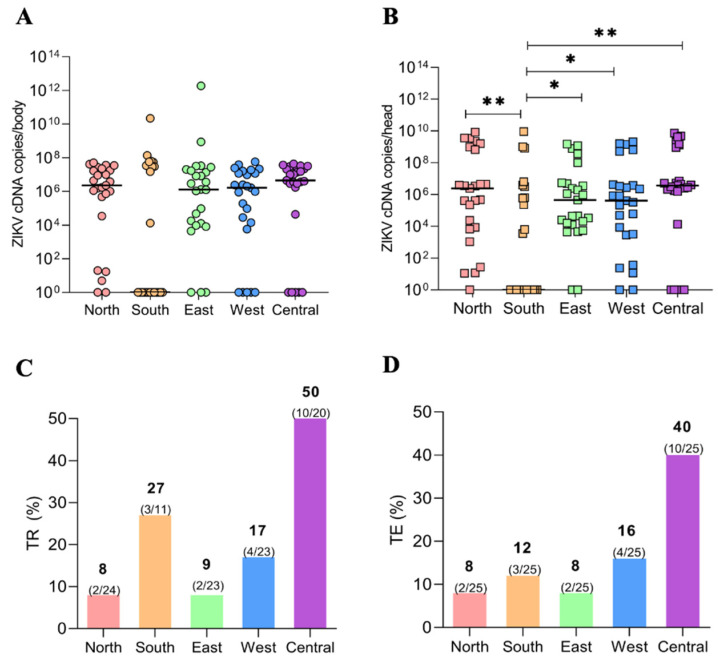
ZIKV viral loads (VLs) per body (**A**) and head/salivary gland **(B**) in *Ae. aegypti* populations from the five districts of Manaus. The VLs were measured as cDNA copies by qPCR at 14 days post-infection. Transmission rate (**C**) and transmission efficiency (**D**) of the 5 *Ae. aegypti* populations from Manaus for ZIKV at 14 days post-infection. The *p* values 0.05 and 0.01 are summarized with one (*) and two (**) asterisks, respectively.

The VLs of ZIKV in the mosquito populations were determined according to the median number of virus cDNA copies in the mosquitoes’ bodies and head/SGs at 14 dpi. In descending order, the median VL of ZIKV cDNA copies in the mosquito bodies were: Central (4 × 10^6^), Northern (2 × 10^6^), Western and Eastern (1 × 10^6^), and Southern district (2 × 10^1^) (Figure 2A). The median ZIKV cDNA copies in the mosquito heads were: Central (3 × 10^6^), Northern (2 × 10^6^), Western and Eastern (4 × 10^5^), and Southern district (4 × 10^2^) (Figure 2B). Comparatively, the ZIKV VLs in the mosquito body were similar among all tested populations (*p* = 0.7252). In contrast, the head/SG VLs varied among populations (*p* = 0.0074), with the Southern population having a smaller value than the others (Figure 2A,B).

### 3.3. Transmission Rate (TR) and Transmission Efficiency (TE)

The mosquito populations were tested for their TR corresponding to the proportion of mosquitoes with infected saliva among mosquitoes with disseminated infection. The TE was determined by the number of samples with infected saliva among the total samples tested. The highest TR value was from the population from the Central district (50%), while the lowest was from the Northern district (8%). The other values in ascending order were from the Eastern (13%), Western (17%), and Southern (27%) districts (Figure 2C).

For the TE, the highest and lowest values were from the populations from the Central (40%) and the Northern (8%) districts. The TE for mosquitoes from the Western district was 16%, and for the Southern and Eastern districts, the values were 12 and 8%, respectively (Figure 2D).

## 4. Discussion

Assessing the patterns of infectivity and dissemination of the arboviruses inside their natural vectors is essential to understand the vector’s susceptibility to these pathogens and the subsequent transmission to the vertebrate hosts. This study evaluated two types of infection parameters: (*i*) the susceptibility of *Ae. aegypti* to the ZIKV infection via an analysis based on the IR (viral ability to infect the mosquito body) and DIR (viral ability to disseminate the infection to secondary organs, such as the salivary glands); and (*ii*) the TR and the efficiency of ZIKV transmission by mosquitoes via an analysis of the secreted saliva. It has already been demonstrated in previous studies that the infection parameters of *Ae. aegypti* after being challenged with an arbovirus can vary in mosquitoes from distinct geographic areas [26], including areas within the same city [2,3], thus corroborating the results presented here for the field-derived populations of *Ae. aegypti* from Manaus.

Concerning the IR and DIR in mosquitoes from the Northern, Eastern, Western, and Central populations, ZIKV was established in a high proportion of infected individuals and disseminated the infection in the tissues while also maintaining high viral loads upon arrival in the salivary glands. This indicates that these mosquitoes are highly permissive to the establishment of ZIKV. In contrast, mosquitoes from the Southern population are less permissive to infection than the other populations, with lower rates of IR and DIR. The high IR and DIR observed in all mosquito populations except the Southern one shows that ZIKV could transpose MIB and MEB in some individuals, disseminate through the hemocoel, and infect secondary organs, such as the salivary glands. For the Southern population, less than half of the mosquitoes challenged with ZIKV were permissive to infection. Variations in the IR and DIR among geographically diverse mosquito populations of the same species can be observed in the literature for different arboviruses in *Aedes* spp. [2,3,16,17,26]. These results are in line with those for the *Ae. aegypti* populations from Belo Horizonte in Brazil [3], with high IR and DIR values for ZIKV infections. A similar vector competence study was conducted in Manaus with four serotypes of DENV and demonstrated that the *Ae. aegypti* populations have variabilities in the IR and DIR, indicating different levels of efficiency in dealing with the virus when considering both infection and dissemination [2].

Concerning the VLs of the five mosquito populations, the similarity in the body values indicates homogeneity in the infection and virus replication in the Northern, Eastern, and Central populations. Moreover, it demonstrates that the individuals with a positive infection from the Southern population had high enough VLs to compensate for the impact of the mosquitoes with a negative one. Additionally, for the VLs in the head/SGs, the lowest value for the Southern population compared with the others may be explained by its low IR. Regarding the five mosquito populations, these VL, IR, and DIR results demonstrate no homogeneity in the virus spreading inside the *Ae. aegypti* body.

After forced salivation, it was possible to detect the mosquitoes with viruses in the saliva and calculate the TR and TE. All *Ae. aegypti* populations from Manaus had individuals that presented ZIKV-positive saliva, demonstrating that the SG barriers have been overcome and that mosquitoes can effectively transmit ZIKV. SG infection is the last step in the mosquito infection cycle. To reach the salivary glands, arboviruses must overcome natural barriers, infect various tissues, and counteract innate and cellular defenses [18]. In the SG, the viruses can be inside the saliva secretory cells after overcoming the SGIB, but, in order to be transmitted to the vertebrate host, they need to cross the SGEB and disseminate to the SG ducts to be present in the mosquito saliva. The TE results showed that even though the Southern population has the lowest IR compared to the others, their TR of 27% (the second highest considering all five populations) allowed it to reach TE rates close to that of the Northern, Eastern, and Western ones. Despite the high IR and DIR, the low TE highlights that SG barriers may prevent virus transmission even if the virus can establish infection and spread in the mosquito body. It shows the importance of assessing all the steps of the viral invasion regarding the infection, dissemination, and reaching of the SGs. The Central population presented high IR, DIR, and TR, thus having the most competent mosquitoes compared to the other ones and demonstrating that, in this population, the ZIKV was successful in all these three steps of viral invasion.

It is known that genetic factors are responsible for most of the characteristics that contribute to the success of the insect vectors, such as susceptibility, vector competence, and insecticide resistance [18]. However, a previous study of population genetics performed with *Ae. aegypti* in Manaus showed no genetic difference between mosquito populations of distinct geographical areas [27]. A potential divergence in the mosquito-inherited microbiota could explain the differences in IR rates, VLs, and TEs since, depending on the species of microorganisms present there and their quantity, it can improve or prevent virus infection, replication, and dissemination in the tissues. Further studies with *Ae. aegypti* from distinct geographical areas of Manaus are necessary to assess whether the composition of their microbiota differs and whether this contributes to their different levels of susceptibility to ZIKV.

Finally, the existence of *Ae. aegypti* populations that are more refractory than others to arbovirus infections in a city, as seen here for the Southern population and for populations in studies carried out in Belo Horizonte for DENV and ZIKV [3] and in Manaus for DENV [2], demonstrate that the mosquito’s susceptibility has the potential to be used in strategies to mitigate arboviral epidemics. It could be used to direct activities to areas where mosquitoes are more competent, in association with the information from the mosquito index. Monitoring city areas and disseminating mosquito competence results to public health agencies may improve the definitions of epidemiological risk areas, thus helping fight the emergence of new outbreaks of arboviral diseases.

## 5. Conclusions

In this study, we observed that all *Ae. aegypti* populations from Manaus have individuals that are competent for transmitting ZIKV. The Southern population stood out for being the least susceptible to ZIKV and supported the least quantity of all, and yet presented equivalent results to the other populations of high infectivity since there was a high proportion of individuals with infected saliva. The Central population was the most competent due to the high proportion of individuals susceptible to ZIKV infection. It was permissive to the virus spreading and crossing the SG cells to reach the saliva. Our results show that determining whether an infected mosquito can transmit an arbovirus is essential to understand the transmission dynamics. They emphasize the importance of analyzing vector–arbovirus interactions for mosquito populations in urban environments. The understanding of the susceptibilities of *Ae. aegypti* mosquitoes from distinct locations to ZIKV, especially in Zika endemic cities, can aid in mitigating arboviral diseases since these data could optimize the definition of city areas that need more efforts to prevent epidemics than others. Complementary studies are therefore necessary to elucidate the factors besides genetics that can interfere with *Ae. aegypti* susceptibility/resistance to ZIKV in geographically close mosquito populations.

## Data Availability

The data that support the findings of this study are available from the corresponding author, (PFPP) upon reasonable request.

## Supplementary Materials

The following supporting information can be downloaded at: https://www.mdpi.com/article/10.3390/v15030770/s1, Figure S1: (A) Negative and positive controls for quantifying ZIKV viral loads (VLs) per body and head/salivary gland. The positive control was a well-established Brazilian colony of Ae. aegypti (strain PP-Campos) susceptible to the ZIKV strain SPH2015; the negative controls were the field-derived mosquitoes fed on uninfected blood. (B) Infection rate (IR), disseminated infection rate (DIR), transmission rate (TR), and transmission efficiency (TE) of the positive control strain of Ae. aegypti.
