# Supplementary material for: Vector Competence for Zika Virus Changes Depending on the Aedes aegypti’s Region of Origin in Manaus: A Study of an Endemic Brazilian Amazonian City
Source: Viruses. 2023 Mar 17;15(3):770. doi: 10.3390/v15030770 (PMC10058289; doi:10.3390/v15030770)
Supplement: Supplementary file 1 [file viruses-15-00770-s001.zip › viruses-2190778-supplementary.pdf]

Supplementary Material

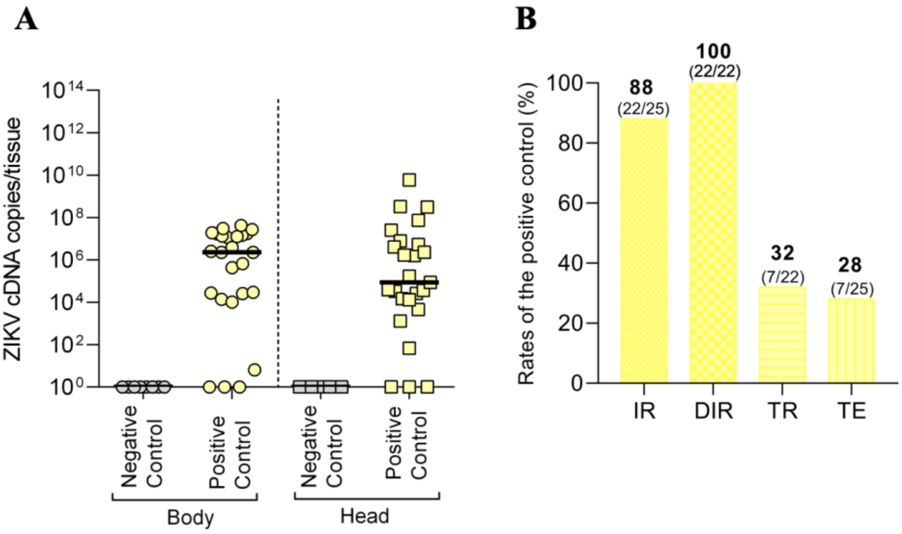

Figure S1. (**A**) Negative and positive controls for quantifying ZIKV viral loads (VLs) per body and head/salivary gland. The positive control was a well-established Brazilian colony of *Ae. aegypti* (strain PP-Campos) susceptible to the ZIKV strain SPH2015; the negative controls were the field-derived mosquitoes fed on uninfected blood. (**B**) Infection rate (IR), disseminated infection rate (DIR), transmission rate (TR), and transmission efficiency (TE) of the positive control strain of *Ae. aegypti*.
